# One-Step Synthesis of Self-Supported Ni_3_S_2_/NiS Composite Film on Ni Foam by Electrodeposition for High-Performance Supercapacitors

**DOI:** 10.3390/nano9121718

**Published:** 2019-12-02

**Authors:** Haifu Huang, Xiaoli Deng, Liqing Yan, Geng Wei, Wenzheng Zhou, Xianqing Liang, Jin Guo

**Affiliations:** 1 Guangxi Novel Battery Materials Research Center of Engineering Technology, Guangxi Colleges and Universities Key Laboratory of Novel Energy Materials and Related Technology, Guangxi Key Laboratory for Relativistic Astrophysics, Center on Nanoenergy Research, School of Physics Science and Technology, Guangxi University, Nanning 530004, China; dengxiaoli@st.gxu.edu.cn (X.D.); 1907301092@st.gxu.edu.cn (L.Y.); 1807301063@st.gxu.edu.cn (G.W.); wzzhou@gxu.edu.cn (W.Z.); lxq@gxu.edu.cn (X.L.); guojin@gxu.edu.cn (J.G.); 2 Guangxi Key Laboratory of Processing for Non-ferrous Metallic and Featured Materials, Guangxi University, Nanning 530004, China

**Keywords:** supercapacitors, nickel sulfide, electrodeposition, nanoflakes

## Abstract

Herein, a facile one-step electrodeposition route was presented for preparing Ni_3_S_2_/NiS composite film on Ni foam substrate (denoted as NiS*_x_*/NF). The NiS*_x_* granular film is composed of mangy interconnected ultra-thin NiS*_x_* nanoflakes with porous structures. When applied as electrodes for supercapacitors, the ultra-thin nanoflakes can provide more active sites for redox reaction, and the interconnected porous structure has an advantage for electrolyte ions to penetrate into the inner space of active materials quickly. As expected, the obtained NiS*_x_*/NF sample exhibited high gravimetric capacitance of 1649.8 F·g^−1^ and areal capacitance of 2.63 F·cm^−2^. Furthermore, a gravimetric capacitance of 1120.1 F·g^−1^ can be maintained at a high current density of 20 mA·cm^−2^, suggesting a good rate capability. The influence of the different molar ratios of electrodeposition electrolyte (NiNO_3_ and thiourea) on the morphology and electrochemical properties of NiS*_x_*/NF sample was investigated to provide an optimum route for one-step electrodeposition of Ni_3_S_2_/NiS composite film. The outstanding performance indicated the Ni_3_S_2_/NiS composite film on Ni foam has great potential as an electrode material for supercapacitors.

## 1. Introduction

At present, the traditional non-renewable fossil energy represented by oil, coal, and natural gas is rapidly consumed, leading to vigorous development of new energy resources (such as solar, wind, and tidal energy). However, these new energy resources are subject to natural environmental conditions. For example, the efficiency of solar energy is limited in rainy and cloudy weather areas. Therefore, the electrochemical energy storage devices connected with them can solve these energy storage and conversion problems [1,2,3,4]. At present, new types of batteries such as lithium-ion batteries, potassium-ion batteries, and lithium–sulfur batteries have the advantage of high energy density, but their low power density and short life cycle limit their application, especially in energy storage systems, which require high-speed and high-power storage devices. Supercapacitors are a new type of energy storage device, which are complementary to batteries because of their high power density and long life cycle, resulting in great advantages in fast energy storage [5,6]. Therefore, energy storage technology based on supercapacitors has attracted much attention.

Currently, supercapacitors have been widely used in aviation, military, vehicle, and electronic devices; they are some of the research focuses in the field of new energy research; but, their energy density is very low, far lower than that of batteries [7]. Among the factors related to energy density, the main component of supercapacitors is electrode materials, so researchers focus on the development of high-performance electrode materials to solve the problem of low energy density [3,8]. In recent years, transition metal sulfides have been widely applied in the field of electrochemistry such as batteries [9], capacitors [10], and the electrochemical detection of glucose [11]. As electrode materials for supercapacitors, transition metal sulfides can provide more electron transport channels and abundant active sites for charge storage. Among various metal sulfides, nickel-based sulfides (NiS, Ni_3_S_2_, and Ni_3_S_4_, etc.) show a great potential as battery-type materials in the application of supercapacitors, due to their high theoretical specific capacitance, abundant resources, and relatively low cost [12]. However, low conductivity is still a prominent disadvantage of the battery-type materials, and nickel-based sulfides are no exception. In addition, these factors, such as the lower electroactive sites and reaction kinetics, also hinder the charge storage performance of nickel sulfide materials. Therefore, nickel sulfide materials exhibit unsatisfactory capacitance performance and rate capability. Building nanostructures is an effective method to improve the charge storage performance of such battery-type materials [13,14,15]. Previous studies have shown that these nanostructures such as nanoparticles [16], nanowires [17], nanorods [18,19], nanoflakes [20,21], and nanoflowers [22] have great advantages in improving performance of nickel sulfide materials. For example, Ni_3_S_2_ nanoparticles prepared by the mechanical alloying method showed high specific capacitance of 911 F·g^−1^ [16]. Furthermore, nanostructures can be combined with self-supporting strategies of direct growth of active materials on the conductive current collector to further optimize the charge storage performance of materials [23,24,25]. Without an additional binder, the active material can be firmly composited on the conductive substrate, which greatly increases the contact surface between the active materials and the conductive substrate, and has a positive effect on the improvement of the conductivity and specific surface area of the active materials. For example, self-supported Ni_3_S_2_ nanosheets array on Ni foam showed a very high capacitance of about 1000 F·g^–1^ [26]. Hierarchical Co_3_O_4_@Ni_3_S_2_ core-shell nanowire arrays on Ni foam exhibited an ultrahigh specific capacitance of 1710 F·g^−1^ at 1 A·g^−1^ [27]. In order to further improve the charge storage capacity of nickel sulfide, it is a common strategy to construct the sulfide compounds of nickel and other metals. For example, Li et al. reported that the specific capacitance of the unique Ni_3_S_2_@CoS core-shell arrays is 4.89 F·cm^−2^ (376.06 F·g^−1^), which is far larger than that of pure Ni_3_S_2_ material (1.97 F·cm^−2^, 164.47 F·g^−1^) [28]. However, it is rarely reported that different crystal structures coexist in the single metal sulfide. Therefore, to effectively enhance the charge storage capacity of nickel-based sulfides, we tried to build a combination of various nanostructured Ni-based sulfides (NiS, Ni_3_S_2_, and Ni_3_S_4_, etc.) as hybrid electrode materials of supercapacitors.

In this work, self-supported Ni_3_S_2_/NiS composite on Ni foam (NiS*_x_*/NF) was prepared by one-step electrodeposition using the cyclic voltammetry method. This electrodeposition method is one with green, low-cost, and scalable properties for preparing electrode nanomaterials. As far as we know, it is the first report about the preparation of Ni_3_S_2_ and NiS composite by the one-step electrodeposition route. In order to optimize the charge storage performance of the as-prepared Ni_3_S_2_/NiS composite materials, we explored the effect of electrodeposition solution concentration on the performance of NiS*_x_*/NF electrode materials. NiS*_x_*/NF was prepared by electrodeposition using different molar ratios of Ni(NO_3_)_2_ and thiourea. The optimum route was obtained by analyzing the morphology, structure, and electrochemical results of the three samples. As expected, the obtained NiS*_x_*/NF sample exhibited remarkable specific capacitance (1649.8 F·g^−1^) and excellent rate capability.

## 2. Experimental Section

### 2.1. Materials and Reagents

Chemical reagent Ni(NO_3_)_2_·6H_2_O and KOH were purchased from Guangdong Guanghua Sci - Tech Co., Ltd. (Guagndong, China), and thiourea was purchased from Afar Sally chemical co. LTD (Tianjin, China). Ni foam (thickness: 1 mm; purity: 99.9%; porosity: 95%; pore size: 0.2–0.5 mm, PPI: 110; and density: 320 g·m^−2^ ± 20) was bought from Shanxi Lizhiyuan Battery Materials Co., Ltd. (Shanxi, China). All the chemical reagents were used as received without further purification.

### 2.2. Preparation of Ni_3_S_2_/NiS Composite on Ni Foam

Ni_3_S_2_/NiS composite (denoted as NiS*_x_*/NF) was electrodeposited into the Ni foam by cyclic voltammetry(CV) method using Gamry electrochemical workstation (Reference 1000, Gamry Instruments). In a typical synthesis, the electrodeposition process was carried out at a scan rate of 5 mV·s^−1^ in the range of −1.2–0.2 V for 30 cycles in a three-electrode system with Ni foam as the working electrode, Ag/AgCl as the reference electrode, and Pt as the counter electrode. The electrodeposition solution was prepared by mixing Ni(NO_3_)_2_·6H_2_O and thiourea in 80 mL H_2_O. The Ni_3_S_2_/NiS composite on Ni foam was prepared using a different solution concentration of Ni(NO_3_)_2_·6H_2_O and thiourea. For better comparison, the molar concentration of thiourea was fixed as 0.5 mol/L^−1^, and the molar concentration of Ni(NO_3_)_2_·6H_2_O was 1, 2.5, and 5 mmol/L^−1^, respectively. The resulting Ni_3_S_2_/NiS composites were denoted as NiS*_x_*/NF-1, NiS*_x_*/NF-2.5, and NiS*_x_*/NF-5, respectively.

### 2.3. Material Characterizations

X-ray diffraction (XRD) patterns were obtained by a Rinku Miniflex 600 diffractometer (Rigaku, Tokyo, Japan). The morphology and elemental composition of as-prepared NiSx/NF were analyzed using a scanning electron microscope (SEM, JSM-6510, JEOL, Tokyo, Japan), field-emission scanning electron microscope (FE-SEM, Zeiss Gemini 500, ZEISS, Oberkochen, Germany), and transmission electron microscope (TEM, Tecnai F20, Philips, Eindhoven, the Netherlands), respectively. 

### 2.4. Electrochemical Measurement

The electrochemical measurements were carried out on a three-electrode system with NiS*_x_*/NF as the working electrode, Hg/HgO as the reference electrode, and Pt sheet as the counter electrode. An amount of 6 M KOH aqueous solution was used as electrolyte. The cyclic voltammetry (CV) was performed at scan rates of 2, 5, 10, 20, 30, 40, and 50 mV·s^−1^ in the range of 0–0.7 V (vs. Hg/HgO) on the Gamry electrochemical workstation (Reference 3000, Gamry Instruments Co., Ltd., Philadelphia, PA, USA), respectively. The galvanostatic charge–discharge (GCD) test was carried out at different current densities of 1, 2, 3, 4 5, 6, 8, 10, 12, 16, and 20 mA·cm^−2^ on the Arbin electrochemical workstation (Arbin Instruments Corp., College Station, Texas, USA), respectively. The electrochemical impedance spectroscopy (EIS) measurement was carried out within a frequency response in the range of 0.01–100 kHz and an AC amplitude of 5 mV on the Gamry electrochemical workstation (Reference 3000).

The galvanostatic capacitance (*C_s_*, F·g^−1^) and areal capacitance (*C_a_*, F·cm^−2^) were calculated using the following formulas:

(1) *C*_*s*_ = (*I△t*)/(*m△V*)

(2) *C*_*a*_ = (*I△t*)/(*s△V*)

where *I*, *△t*, *△V*, *m*, and *S* are the discharge current (A), the discharge time (s), the potential voltage (V), the total mass of the active materials (g), and the geometric area of electrode, respectively.

## 3. Results and Discussion

### 3.1. Characteristics 

**Figure 1:**
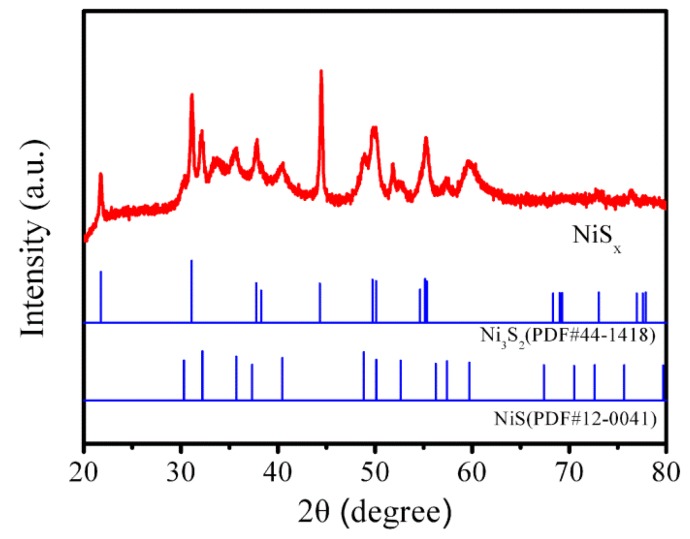
The XRD patterns of the NiS*_x_* powder.

The XRD patterns of the NiS*_x_* powder obtained from NiSx/NF electrode are shown in Figure 1. The diffraction peaks are well-matched with both Ni_3_S_2_ planes (PDF#44-1418) and NiS phase (PDF# 12-0041), confirming that the mixed phase of Ni_3_S_2_ and NiS was formatted in the as-prepared NiS*_x_* composite film. The impurity peaks at 51.8° are attributed to the (200) crystal planes of residual nickel metal (PDF# 04-0850) from Ni foam.

**Figure 2:**
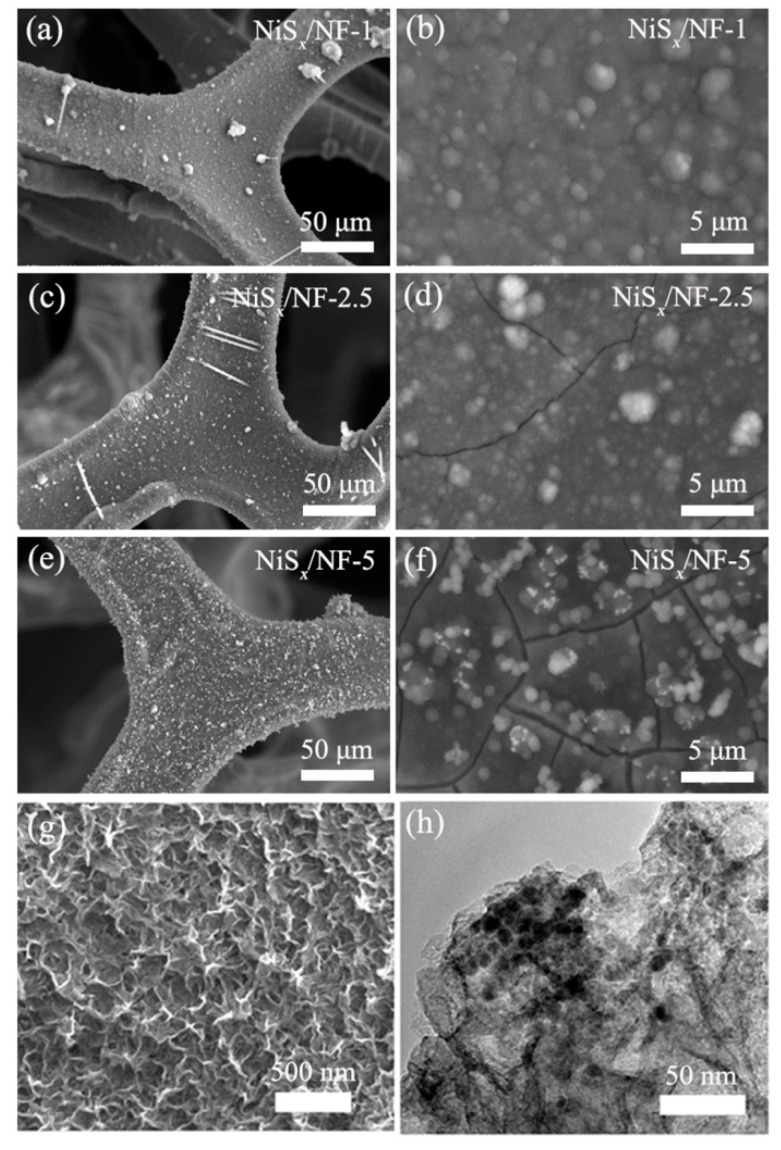
SEM images: (**a**–**b**) NiS*_x_*/NF-1, (**c**–**d**) NiS*_x_*/NF-2.5, and (**e**–**f**) NiS*_x_*/NF-5; (**g**) FE-SEM image of NiS*_x_*/NF; and (**h**) TEM image.

**Figure 3:**
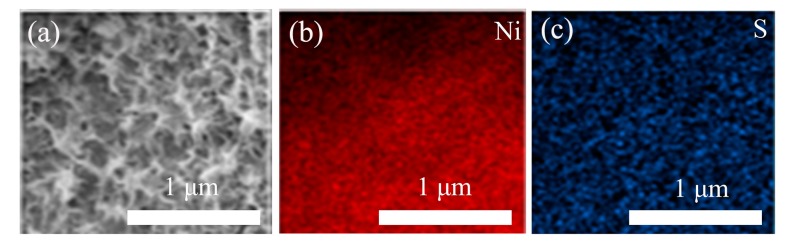
(**a**) the SEM image, and the corresponding elemental mapping (**b**) Ni, (**c**) S.

Figure 2 shows the SEM images of NiS*_x_* composite on Ni foam prepared using different molar ratios of NiNO_3_ and thiourea electrolyte. In the low-magnification SEM images (Figure 2a,c,e), NiS*_x_* has uniformly deposited on the framework of the Ni foam. Further, NiS*_x_* composite thin films showed granular morphology, confirmed by the SEM images at high magnification (Figure 2b,d,f). As the molar concentration of Ni(NO_3_)_2_ increased, the thickness and the wrinkle degree of NiS*_x_* film also increased. Meanwhile, some microspheres on the surface of NiS*_x_* composite films were observed. The increase of microspheres can provide more active sites for redox reaction, resulting in high specific capacitance of NiS*_x_* composite. It is worth noting that large cracks gradually appear in the film when concentration of NiNO_3_ electrolyte increases. Therefore, the tightness of the film is reduced with the increase of Ni(NO_3_)_2_ molar concentration. The expanding transverse crack may cause the NiS*_x_* composite film to fall off, resulting in a rapid decline in the capacitance of NiS*_x_* composite electrode after repeating charge–discharge tests. More detailed morphology was further observed by field-emission scanning electron microscopy (FE-SEM) and a transmission electron microscope (TEM), as shown in Figure 2g–h. The FE-SEM image (Figure 2g) shows that the granular morphology was composed of mangy interconnected ultra-thin NiS*_x_* composite nanoflakes with porous structures, which is advantageous for electrolyte to penetrate into the inner space of active materials quickly. The reasons for the increase of wrinkle degree of NiS*_x_* film may be as follows. When the concentration of electrodeposition solution is relatively low, the as-prepared films should be relatively dense. As the arrays of supported NiS*_x_* nanoflakes gradually form, the film has porous characteristics, so the film will become more and more rough. The TEM image (Figure 2h) further confirms the ultra-thin NiS*_x_* composite nanoflakes were overlapping and interconnected. The energy-dispersive X-ray spectroscopy (EDX) mapping was further employed to investigate the elemental distribution of Ni and S. As shown in Figure 3, elements Ni and S were uniformly distributed, confirming the successful deposition of the NiS*_x_* nanosheets.

### 3.2. Electrochemical Performance

**Figure 4:**
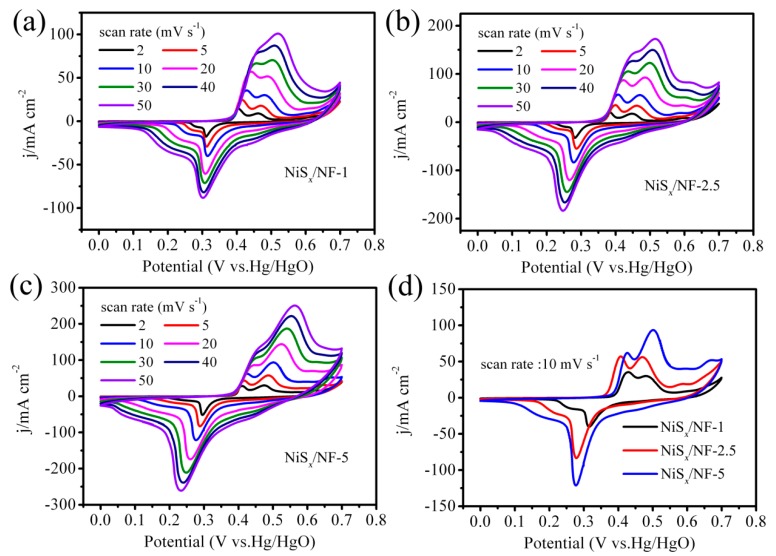
CV curves: (**a**) NiS*_x_*/NF-1, (**b**) NiS*_x_*/NF-2.5, and (**c**) NiS*_x_*/NF-5; and (**d**) the comparison of CV curves between three samples at the same scanning rate.

The electrochemical performance of samples NiS*_x_*/NF-1, NiS*_x_*/NF-2.5, and NiS*_x_*/NF-5 were evaluated by a three-electrode system. Figure 4a–c shows the typical CV curves of samples. It can be seen that there are well-defined redox peaks at different scan rates from 2 to 50 mV·s^−1^, which indicates the Faraday reaction nature of the NiS*_x_*/NF electrode [29]. The redox reaction of nickel sulfides in alkaline electrolyte is expressed as shown below [30,31,32].

(3) Ni_3_S_2_ + 3OH^−^ ↔ Ni_3_S_2_(OH)_3_ + 3e^−^

(4) NiS + OH^−^ ↔ NiSOH + e^−^

When the scan rate increased, the corresponding current increased, and the redox peaks moved to both sides due to an enhanced polarization at high scan rate. Meanwhile, active materials failed to fully contact with ions at high scan rate, resulting in a reduction in the number of active site for redox reactions, so it can be observed there were some changes in the shape of CV curves with the increased scan rates. Figure 4d shows the comparison of the CV curves of three samples (NiS*_x_*/NF-1, NiS*_x_*/NF-2.5, and NiS*_x_*/NF-5) at the same scan rate of 10 mV·s ^−1^. Usually, the area enclosed by CV curves can reflect the specific capacitance (gravimetric capacitance or areal capacitance) of active materials. Herein, the sample NiS*_x_*/NF-5 shows the largest area enclosed by CV curves between three samples, suggesting that it has the highest areal specific capacitance.

**Figure 5:**
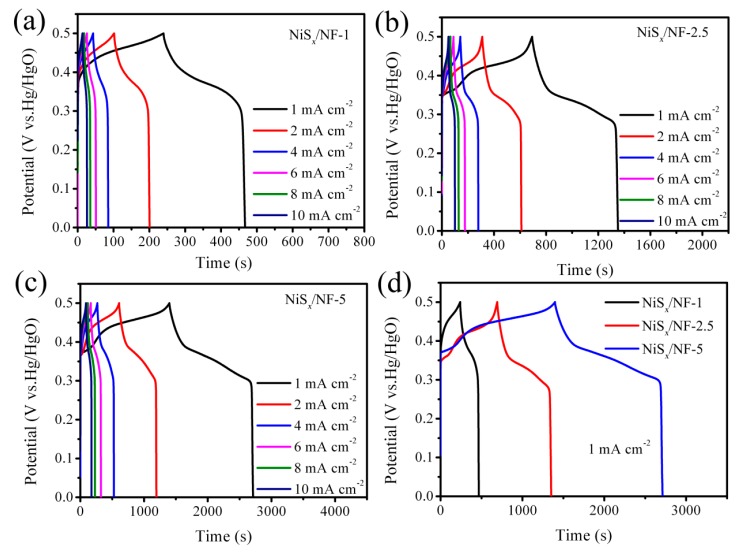
The galvanostatic charge–discharge(GCD)curves: (**a**) NiS*_x_*/NF-1, (**b**) NiS*_x_*/NF-2.5, and (**c**) NiS*_x_*/NF-5; (**d**) the comparison of GCD curves between three samples at the same current density.

**Figure 6:**
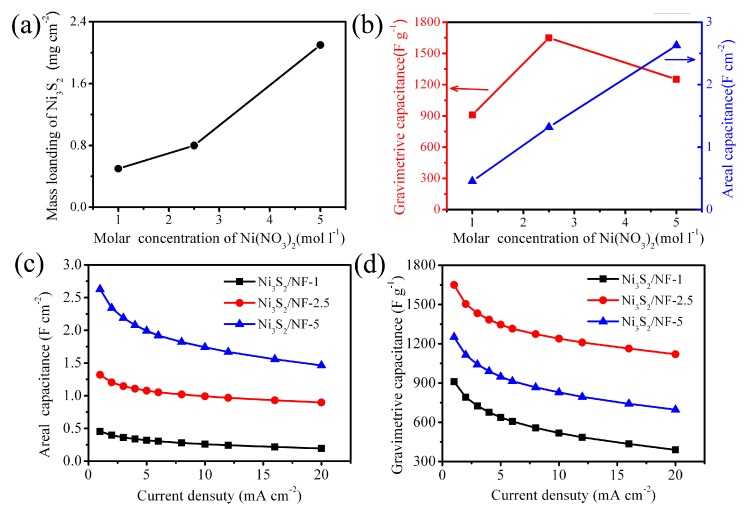
(**a**) The mass loading of as-prepared NiS*_x_* on Ni foam. (**b**) The areal capacitance and gravimetric capacitance of as-prepared NiS*_x_*/NF at the same current density of 1 mA·cm^−2^ for comparison. (**c**–**d**) The areal and gravimetric capacitance of NiS*_x_*/NF electrodes as a function of charge−discharge current densities.

The galvanostatic charge–discharge (GCD) tests were carried out at different current densities ranging from 1 to 20 mA·cm^−2^. Typical GCD curves at current densities of 1, 2, 4, 6, 8, and 10 mA·cm^−2^ are shown in Figure 5. It was revealed that the GCD curves of those three samples were all non-linear, further indicating that the energy storage of the NiS*_x_*/NF electrode came from the Faraday reaction. Figure 5d exhibits the GCD curves at the same current density of 1 mA·cm^−2^ for comparison. The corresponding mass loading, areal capacitance, and gravimetric capacitance of as-prepared NiS*_x_*/NF are provided in Figure 5a,b. Obviously, areal capacitance increases from 0.46 to 2.63 F·cm^−2^ with the increase of molar concentration of NiNO_3_. The NiS*_x_*/NF-5 sample has the highest areal capacitance of 2.63 F·cm^−2^, while NiS*_x_*/NF-2.5 exhibits the highest gravimetric capacitance of 1649.8 F·g^−1^. The areal and gravimetric capacitance of NiS*_x_*/NF electrodes as a function of charge−discharge current densities are summarized in Figure 6c,d. Due to the influence of electrodeposition solution, the specific capacitance shows a great difference. The possible explanation is as follows**.** Under the same cycle number of electrodeposition, it is obvious that the mass loading and thickness of NiS*_x_* (Ni_3_S_2_/NiS) film coated on the Ni foam will increase when the molar concentration of Ni(NO_3_)_2_ increases, as shown in Figure 2a–f and Figure 6a. It can also promote the increase of areal capacitance. At the same time, by comparing the morphologies of NiS*_x_*/NF-1, NiS*_x_*/NF-2.5, and NiS*_x_*/NF-5, it found that the porous structure and the degree of wrinkle of the film increase with the increase of the concentration, which means that the specific surface area of the active electrode materials will increase, and more active points will be exposed to the electrolyte solution. In addition, the microspheres anchored on NiS*_x_* film increase gradually as the molar concentration of Ni(NO_3_)_2_ increases, which further increases the utilization of active materials. Therefore, the specific capacitance should be enhanced. However, when the thickness and mass loading of the deposited NiS*_x_* film are excessive, there is no doubt that it is difficult for electrolyte to penetrate into the inner area of the electrode materials, which leads to the low effective utilization of the active materials. Therefore, it is natural that the mass specific capacitance will be reduced. 

Compared with other reported materials, the material prepared by our work has obvious performance advantages. The specific capacitance value was higher than those of previous nickel sulfide-based materials as supercapacitor electrodes, for instance: nanosheet-based Ni_3_S_2_ microspheres on Ni foam (981.8 F·g^−1^) [33], porous NiS nanoflake arrays (718 F·g^−1^) [34], Ni_3_S_2_ on Ni foam with rGO (1462 F·g^−1^) [35], 3D graphene/Ni_3_S_2_ (741 F·g^−1^) [36], 3D reduced graphene oxide wrapped Ni_3_S_2_ nanoparticles on Ni Foam (816.8 F·g^−1^) [37], Ni_3_S_2_@β-NiS materials (1158 F·g^−1^) [32], and graphene-coupled flower-like Ni_3_S_2_ (1315 F·g^−1^) [38]. The enhanced specific capacitance should be attributed to the porous structure formed by interconnected ultra-thin nanoflakes and synergistic effect between Ni_3_S_2_ and NiS.

**Figure 7:**
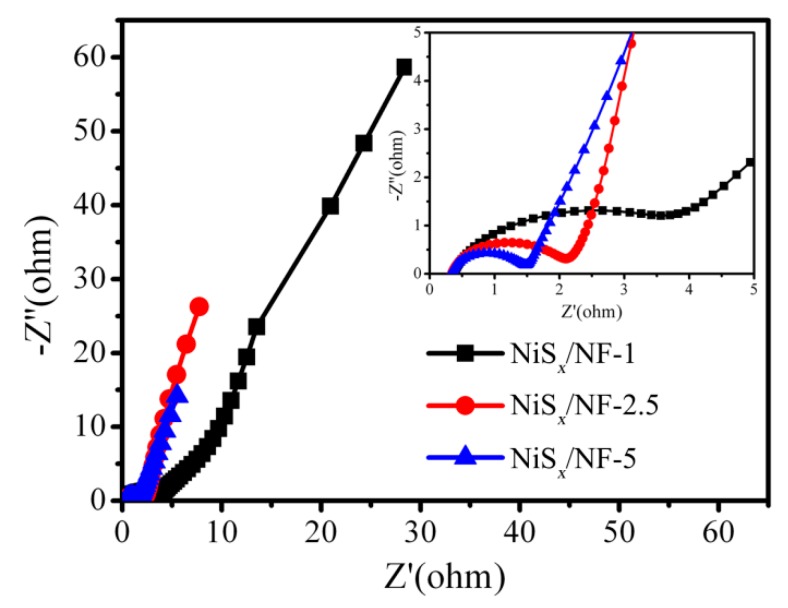
The Nyquist curves of three samples.

The electrochemical impedance spectroscopy (EIS) of the as-prepared NiS*_x_*/NF-1, NiS*_x_*/NF-2.5, and NiS*_x_*/NF-5 was used to study the intrinsic electrochemical behavior, and corresponding Nyquist curves are shown in Figure 7. In the low-frequency region, all curves exhibit a straight line, indicating capacitive behavior. In the high-frequency region, all curves exhibit a small semicircle. The semicircle is related to the electrode surface properties, and the corresponding diameter represents the value of charge–transfer resistance (R_ct_). The diameter of the semicircle for the samples (NiS*_x_*/NF-1, NiS*_x_*/NF-2.5, and NiS*_x_*/NF-5) decreases gradually, meaning that charge transfer and ion transfer rate have been improved. Further, the intercept of Nyquist curve at the Z’ axis represents equivalent series resistance (ESR). The ESR values of the NiS*_x_*/NF-1, NiS*_x_*/NF-2.5, and NiS*_x_*/NF-5 electrodes are 0.36, 0.34, and 0.33 Ω, respectively. It also indicates the contact resistance at the interface of the NiS*_x_* film and Ni foam was very low.

**Figure 8:**
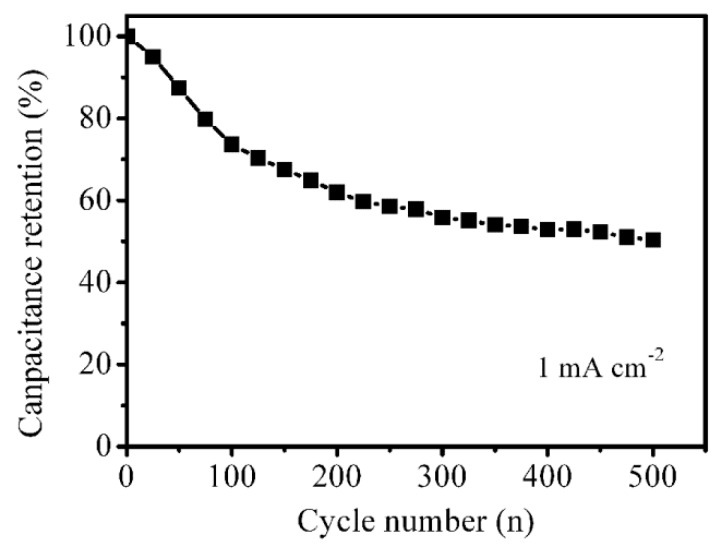
The evaluation of the cycle stability.

The cycle stability was evaluated by repeating the charge–discharge test for 500 cycles, as shown in Figure 8. In repeated charging and discharging cycles, the capacitance of the NiS*_x_*/NF electrode decreased gradually. After 500 cycles, the capacitance decreased to 50% of the original capacitance, suggesting that the cycle stability of NiS*_x_*/NF electrode is not good. Based on SEM images, the cracks in the NiS*_x_* film on Ni foam can be observed. The repeated charge and discharge process may cause the loose connection between NiS*_x_* and Ni foam, and then NiS*_x_* drops off from Ni foam, resulting in the decrease of the capacitance of the NiS*_x_*/NF electrode materials.

## 4. Conclusions

In this paper, the mixed Ni_3_S_2_/NiS composite (NiS*_x_*/NF) was prepared by electrodeposition on the Ni foam by cyclic voltammetry. Then, the influence of the different molar ratios of NiNO_3_ and thiourea electrolyte on the morphology and electrochemical properties of NiS*_x_*/NF electrode was investigated. The granular morphology and interconnected ultra-thin nanoflakes presented in NiS*_x_* thin films can provide more active sites for redox reaction, resulting in high specific capacitance of NiS*_x_*/NF. As-obtained NiS*_x_*/NF exhibits a high gravimetric capacitance up to 1649.8 F·g^−1^ at 1 mA·cm^−2^. Meanwhile, areal capacitance of 2.63 F·cm^−2^ can be achieved, demonstrating great application potential of Ni_3_S_2_/NiS composite.

## References

[1] Yang Z., Zhang J., Kintner-Meyer M.C., Lu X., Choi D., Lemmon J.P., Liu J. (2011). Electrochemical energy storage for green grid. Chem. Rev..

[2] Goodenough J.B. (2014). Electrochemical energy storage in a sustainable modern society. Energy Environ. Sci..

[3] Shao Y., El-Kady M.F., Sun J., Li Y., Zhang Q., Zhu M., Wang H., Dunn B., Kaner R.B. (2018). Design and mechanisms of asymmetric supercapacitors. Chem. Rev..

[4] Wang L., Zhang Q., Zhu J., Duan X., Xu Z., Liu Y., Yang H., Lu B. (2019). Nature of extra capacity in MoS_2_ electrodes: Molybdenum atoms accommodate with lithium. Energy Storage Mater..

[5] Huang H., Lei C., Luo G., Li G., Liang X., Tang S., Du Y. (2016). UV-assisted reduction of graphene oxide on Ni foam as high performance electrode for supercapacitors. Carbon.

[6] Feng Y., Chen S., Wang J., Lu B. (2020). Carbon foam with microporous structure for high performance symmetric potassium dual-ion capacitor. J. Energy Chem..

[7] Fan L., Ma R., Zhang Q., Jia X., Lu B. (2019). Graphite Anode for Potassium Ion Battery with Unprecedented Performance. Angew. Chem. Int. Ed..

[8] Wang J., Dong S., Ding B., Wang Y., Hao X., Dou H., Xia Y., Zhang X. (2016). Pseudocapacitive materials for electrochemical capacitors: From rational synthesis to capacitance optimization. Nat. Sci. Rev..

[9] Yu Z., Kang Z., Hu Z., Lu J., Zhou Z., Jiao S. (2016). Hexagonal NiS nanobelts as advanced cathode materials for rechargeable Al-ion batteries. Chem. Commun..

[10] Radhakrishnan S., Kim H.Y., Kim B.S. (2016). Expeditious and eco-friendly fabrication of highly uniform microflower superstructures and their applications in highlydurable methanol oxidation and high-performance supercapacitors. J. Mater. Chem. A.

[11] Radhakrishnan S., Kim H.Y. (2015). Facile fabrication of NiS and reduced graphene oxide hybrid film for nonenzymatic detection of glucose. RSC Adv..

[12] Zhang C., Huang Y., Tang S., Deng M., Du Y. (2017). High-energy all-solid-state symmetric supercapacitor based on Ni_3_S_2_ mesoporous nanosheet-decorated three-dimensional reduced graphene oxide. ACS Energy Lett..

[13] Huang H., Zhang H., Fan Y., Deng X., Li G., Liang X., Zhou W., Guo J., Tang S. (2019). Serrated-like NiCoO_2_ nanoarrays on Ni foam for high-performance supercapacitors. Appl. Surf. Sci..

[14] Naoi K., Naoi W., Aoyagi S., Miyamoto J.-I., Kamino T. (2012). New generation “nanohybrid supercapacitor”. Acc. Chem. Res..

[15] Liu T., Jiang C., Cheng B., You W., Yu J. (2017). Hierarchical NiS/N-doped carbon composite hollow spheres with excellent supercapacitor performance. J. Mater. Chem. A.

[16] Li J.-J., Hu Y.-X., Liu M.-C., Kong L.-B., Hu Y.-M., Han W., Luo Y.-C., Kang L. (2016). Mechanical alloying synthesis of Ni_3_S_2_ nanoparticles as electrode material for pseudocapacitor with excellent performances. J. Alloys Compds..

[17] Xu S., Wang T., Ma Y., Jiang W., Wang S., Hong M., Hu N., Su Y., Zhang Y., Yang Z. (2017). Cobalt Doping to Boost the Electrochemical Properties of Ni@Ni_3_S_2_ Nanowire Films for High-Performance Supercapacitors. ChemSusChem.

[18] Yang J., Duan X., Guo W., Li D., Zhang H., Zheng W. (2014). Electrochemical performances investigation of NiS/rGO composite as electrode material for supercapacitors. Nano Energy.

[19] Qu C., Zhang L., Meng W., Liang Z., Zhu B., Dang D., Dai S., Zhao B., Tabassum H., Gao S. (2018). MOF-derived α-NiS nanorods on graphene as an electrode for high-energy-density supercapacitors. J. Mater. Chem. A.

[20] Krishnamoorthy K., Veerasubramani G.K., Radhakrishnan S.,  Kim S.J. (2014). One pot hydrothermal growth of hierarchical nanostructured Ni_3_S_2_ on Ni foam for supercapacitor application. Chem. Engine J..

[21] Yu L., Yang B., Liu Q., Liu J., Wang X., Song D., Wang J., Jing X. (2015). Interconnected NiS nanosheets supported by nickel foam: Soaking fabrication and supercapacitors application. J. Electroanal. Chem..

[22] Ghosh D., Das C.K. (2015). Hydrothermal growth of hierarchical Ni_3_S_2_ and Co_3_S_4_ on a reduced graphene oxide hydrogel@ Ni foam: A high-energy-density aqueous asymmetric supercapacitor. ACS Appl. Mater. Interfaces.

[23] Deng X., Fan Y., Zhou Q., Huang H., Zhou W., Lan Z., Liang X., Li G., Guo J., Tang S. (2019). Self-supported Ni_3_S_2_/NiCo_2_O_4_ core-shell flakes-arrays on Ni foam for enhanced charge storage properties. Electrochim. Acta.

[24] Zhang G., Lou X.W. (2013). General solution growth of mesoporous NiCo_2_O_4_ nanosheets on various conductive substrates as high-performance electrodes for supercapacitors. Adv. Mater..

[25] Chen Y., Qu B., Hu L., Xu Z., Li Q., Wang T. (2013). High-performance supercapacitor and lithium-ion battery based on 3D hierarchical NH_4_F-induced nickel cobaltate nanosheet–nanowire cluster arrays as self-supported electrodes. Nanoscale.

[26] Chen J.S., Guan C., Gui Y., Blackwood D.J. (2017). Rational Design of Self-Supported Ni_3_S_2_ Nanosheets Array for Advanced Asymmetric Supercapacitor with a Superior Energy Density. ACS Appl. Mater. Interfaces.

[27] Zhang J., Lin J., Wu J., Xu R., Lai M., Gong C., Chen X., Zhou P. (2016). Excellent Electrochemical Performance Hierarchical Co_3_O_4_@Ni_3_S_2_ core/shell nanowire arrays for Asymmetric Supercapacitors. Electrochim. Acta.

[28] Li R., Wang S., Wang J., Huang Z. (2015). Ni_3_S_2_@CoS core–shell nano-triangular pyramid arrays on Ni foam for high-performance supercapacitors. Phys. Chem. Chem. Phys..

[29] Brousse T., Bélanger D., Long J.W. (2015). To be or not to be pseudocapacitive?. J. Electrochem. Soc..

[30] Zhang Z., Zhao C., Min S., Qian X. (2014). A facile one-step route to RGO/Ni3S2 for high-performance supercapacitors. Electrochim. Acta.

[31] Xing Z., Chu Q., Ren X., Ge C., Qusti A.H., Asiri A.M., Al-Youbi A.O., Sun X. (2014). Ni_3_S_2_ coated ZnO array for high-performance supercapacitors. J. Power Sources.

[32] Li W., Wang S., Xin L., Wu M., Lou X. (2016). Single-crystal β-NiS nanorod arrays with a hollow-structured Ni_3_S_2_ framework for supercapacitor applications. J. Mater. Chem. A.

[33] Li G., Cong Y., Zhang C., Tao H., Sun Y., Wang Y. (2017). Hierarchical nanosheet-based Ni_3_S_2_ microspheres grown on Ni foam for high-performance all-solid-state asymmetric supercapacitors. Nanotechnology.

[34] Yan X., Tong X., Ma L., Tian Y., Cai Y., Gong C., Zhang M., Liang L. (2014). Synthesis of porous NiS nanoflake arrays by ion exchange reaction from NiO and their high performance supercapacitor properties. Mater. Lett..

[35] Lv J., Liang T., Yang M., Suzuki K., Miura H. (2017). The plume-like Ni_3_S_2_ supercapacitor electrodes formed on nickel foam by catalysis of thermal reduced graphene oxide. J. Electroanal. Chem..

[36] Wu P., Wang D., Ning J., Zhang J., Feng X., Dong J., Hao Y. (2018). Novel 3D porous graphene/Ni_3_S_2_ nanostructures for high-performance supercapacitor electrodes. J. Alloys Compds..

[37] Qi J., Chang Y., Sui Y., He Y., Meng Q., Wei F., Zhao Y., Jin Y. (2017). Facile Construction of 3D Reduced Graphene Oxide Wrapped Ni_3_S_2_ Nanoparticles on Ni Foam for High-Performance Asymmetric Supercapacitor Electrodes. Part. Part. Syst. Char..

[38] Lin H., Liu F., Wang X., Ai Y., Yao Z., Chu L., Han S., Zhuang X. (2016). Graphene-Coupled Flower-Like Ni_3_S_2_ for a Free-Standing 3D Aerogel with an Ultra-High Electrochemical Capacity. Electrochim. Acta.

